# What Are the Peripheral Blood Determinants for Increased Osteoclast Formation in the Various Inflammatory Diseases Associated With Bone Loss?

**DOI:** 10.3389/fimmu.2019.00505

**Published:** 2019-03-19

**Authors:** Teun J. de Vries, Ismail el Bakkali, Thomas Kamradt, Georg Schett, Ineke D. C. Jansen, Patrizia D'Amelio

**Affiliations:** ^1^Department of Periodontology, Academic Centre for Dentistry Amsterdam (ACTA), University of Amsterdam and Vrije Universiteit Amsterdam, Amsterdam, Netherlands; ^2^Institute of Immunology, Universitätsklinikum Jena, Jena, Germany; ^3^Department of Internal Medicine III, Friedrich-Alexander University Erlangen-Nürnberg and Universitatsklinikum Erlangen, Erlangen, Germany; ^4^Gerontology and Bone Metabolic Diseases Division, Department of Medical Science, University of Turin, Turin, Italy

**Keywords:** peripheral blood, osteoclast formation, T-cells, CD14+CD16+, osteoclast precursor priming, inflammatory bone diseases

## Abstract

Local priming of osteoclast precursors (OCp) has long been considered the main and obvious pathway that takes place in the human body, where local bone lining cells and RANKL-expressing osteocytes may facilitate the differentiation of OCp. However, priming of OCp away from bone, such as in inflammatory tissues, as revealed in peripheral blood, may represent a second pathway, particularly relevant in individuals who suffer from systemic bone loss such as prevalent in inflammatory diseases. In this review, we used a systematic approach to review the literature on osteoclast formation in peripheral blood in patients with inflammatory diseases associated with bone loss. Only studies that compared inflammatory (bone) disease with healthy controls in the same study were included. Using this core collection, it becomes clear that experimental osteoclastogenesis using peripheral blood from patients with bone loss diseases in prevalent diseases such as rheumatoid arthritis, osteoporosis, periodontitis, and cancer-related osteopenia unequivocally point toward an intrinsically increased osteoclast formation and activation. In particular, such increased osteoclastogenesis already takes place without the addition of the classical osteoclastogenesis cytokines M-CSF and RANKL *in vitro*. We show that T-cells and monocytes as OCp are the minimal demands for such unstimulated osteoclast formation. In search for common and disease-specific denominators of the diseases with inflammation-driven bone loss, we demonstrate that altered T-cell activity and a different composition—such as the CD14+CD16+ vs. CD14+CD16– monocytes—and priming of OCp with increased M-CSF, RANKL, and TNF- α levels in peripheral blood play a role in increased osteoclast formation and activity. Future research will likely uncover the barcodes of the OCp in the various inflammatory diseases associated with bone loss.

## Introduction

Close inspection of skeletons as seen in anatomy museums may show signs of inflammatory bone loss, present as joint erosions and bone degradation of the tooth sockets that surround teeth. This betrays an evoked activity of bone degradation by inflammation steered osteoclasts, so different from turn-over osteoclasts that in fact leave the rest of the skeleton seemingly normal. The different bone cells, osteoclasts, bone-lining cells, osteoblasts and osteocytes are responsible for a lifelong balanced remodeling process. When this process becomes unbalanced, such as during inflammatory diseases with bone-loss, it may result in severe bone loss, locally, or systemically. The key cell-type in this disturbed bone balance is the osteoclast, the multinucleated cell responsible for breaking down bone tissue (1).

Osteoclasts derive from cells of the monocyte/macrophage lineage (2–4) present in bone marrow (5), but also present in peripheral blood (6). Osteoclasts play a key role in diseases that are associated with increased bone loss (7). Such diseases include common diseases such as the rheumatic diseases, osteoporosis, periodontitis, cancers that metastasize to bone and Crohn's disease. Less common diseases that also give rise to bone loss are chronic liver disease, Gaucher's disease, Turner's syndrome, and phenylketonuria. Bone loss is also frequently observed in patients with chronic kidney disease. In nearly all of these diseases, excessive osteoclast generation and activation, with a key contribution to the altered immune system plays a dominant role. All these diseases are discussed in detail below.

Growth factors and cytokines that can be produced by a wide range of cells in the human body regulate the activity and formation of osteoclasts. The principle differentiation factors for osteoclast differentiation are macrophage colony-stimulating factor (M-CSF), receptor activator of nuclear factor kappa-B ligand (RANKL) (8) and the inhibitor of osteoclast differentiation, the “protector of bone,” osteoprotegerin (OPG) (9). These cytokines are produced by osteocytes and bone lining cells/osteoblasts (10). However, RANKL can also be produced by T-cells (11, 12), by synovial fibroblasts from inflamed joints (13) and by tooth-associated fibroblasts (14). Apart from this classical pathway, it was demonstrated by Kim et al. using RANK-/- mouse models, that osteoclasts may also form through stimulation with inflammatory cytokines such as tumor necrosis factor-α (TNF-α) (15).

Apart from the common culture methods of osteoclasts using M-CSF and RANKL, there are strong indications that osteoclast precursors may differentiate into multinucleated osteoclasts in the absence of added osteoclastogenesis stimulating factors M-CSF and RANKL. This is referred to as “spontaneous” or unstimulated osteoclast formation (16, 17), recently excellently reviewed by Salamanna et al. (7). A better term for this could be self-stimulatory osteoclastogenesis, where the combination of T-cells that may provide the osteoclastogenesis signals with primed OCp may give rise to osteoclast formation. Here, and experimental evidence is provided below, cells from peripheral blood, such as T-cells, may provide the necessary differentiation factors for the monocytic, CD14+ osteoclast precursor cells in blood. These studies are based on experimental *in vitro* studies which suggest an activation of the OCp by inflammatory mediators present in the plasma of patients with inflammatory bone disease or an intrinsic change of cells toward more osteoclastic differentiation. Examples include periodontitis, osteoporosis, Crohn's disease, rheumatoid arthritis, and bone metastatic cancer and will be further described later on in this review.

The aim of this systematic literature review is to provide an overview and an interpretation of experimental *in vitro* studies involving osteoclast formation from peripheral blood of patients with inflammatory diseases that lead to bone loss compared to the osteoclast formation from peripheral blood of healthy controls. This will gain insight into the various mechanisms that play a role in the activation of osteoclasts from peripheral blood in these inflammatory bone diseases. The similarities and differences in peripheral blood-mediated osteoclast formation between the various inflammatory diseases associated with bone loss will be examined and discussed.

## Literature Search

The methodological approach of systematic review was used. The PRISMA (Preferred Reporting Items for Systematic Reviews and Meta-Analyses) was used to reduce the bias in the selection of publications for this review.

Search strategy

Search Query

#1 Peripheral blood
#2 Osteoclast OR osteoclasts
#3 Osteoclast formation OR osteoclast differentiation OR osteoclastogenesis
#4 (Periodontitis OR periodontal disease) OR rheumatoid arthritis OR psoriatic arthritis OR inflammatory bowel disease OR Crohn's disease OR osteoporosis OR bone metastatic cancer
#5 (#1 AND #2 AND #3 AND #4) AND (“2002/01/01”[PDat]: “2018/07/31”[PDat])

The electronic search for relevant studies was carried out in the three databases Pubmed, Embase, and Web of science. The keywords used for the search were “peripheral blood,” “osteoclast,” “osteoclasts,” “osteoclast formation,” “osteoclast differentiation,” “osteoclastogenesis,” “periodontitis,” “periodontal disease,” “rheumatoid arthritis,” “psoriatic arthritis,” “inflammatory bowel disease,” “Crohn's disease,” “osteoporosis,” “bone metastatic cancer.” In terms of time and language, articles published from 2002 until 2018 were assessed and only publications in English were included in this study.

## Screening and Selection

A set of inclusion and exclusion criteria were used to retrieve relevant *in vitro* studies. All *in vitro* studies involving exogenously added cytokine driven and spontaneous osteoclast formation from peripheral blood from patients with periodontitis or rheumatoid arthritis or psoriatic arthritis or osteoporosis or Crohn's disease or bone metastatic cancer were included for further examination.

**Figure 1:**
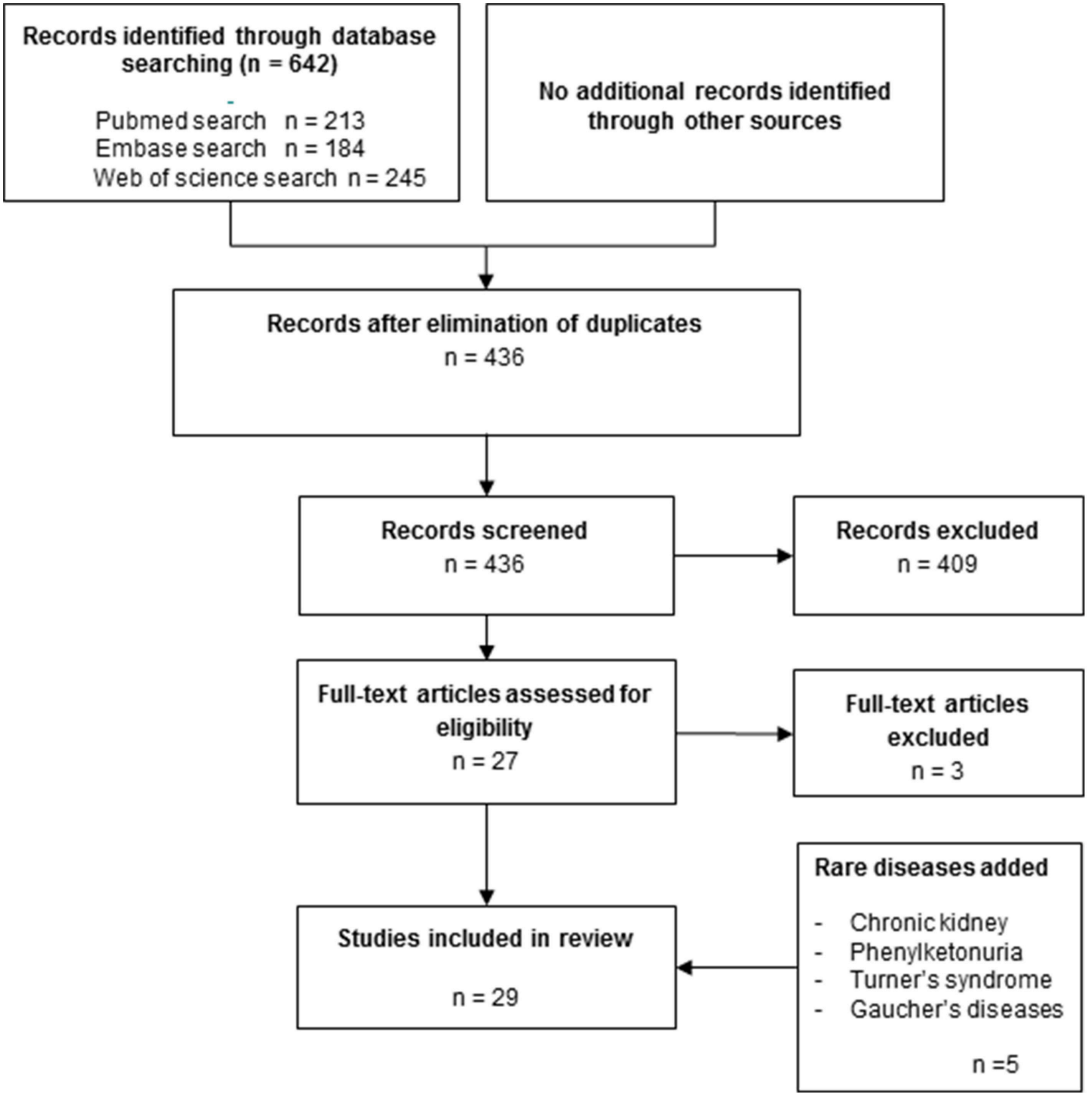
Flow chart of the literature search strategy.

The titles and abstracts of all publications identified by the electronic search were manually screened and discussed by two reviewers (IB and TdV). When the suitability of an article for this review could not be determined based on the title and abstract, the full text was read and examined by one reviewer (IB) and reported and discussed (IB and TdV). Outcomes of *in vitro* studies included comparison between osteoclast formation from peripheral blood of patients with one of the diseases listed above and healthy controls. All articles that did not meet with the primary outcome of interest, (spontaneous) osteoclast formation from PBMCs or monocytes of diseased patients compared to healthy controls were excluded, leaving 29 papers in the core-collection (Figure 1).

Apart from the systematic approach on the common diseases osteoporosis, periodontitis and rheumatoid arthritis, literature on less frequent diseases (Turner, Gaucher, chronic liver disease, Crohn's disease, and phenylketonuria) that was found with this search strategy was incorporated.

## Peripheral Blood Osteoclast Formation Is Increased in a Wide Spectrum of Bone Diseases

Overall it can be concluded that osteoclast formation from peripheral blood cells is increased in a wide range of diseases (Table 1). Results per disease or group of diseases are discussed in detail below.

**Table 1:** Unstimulated and stimulated osteoclast formation is increased in peripheral blood from patients with bone loss.

	**Unstimulated [without cytokines]**			**Stimulated [M-CSF + RANKL]**		
**Disease, study**	**Disease**	**Healthy control**	**Resorption?**	**Disease**	**Healthy control**	**Resorption?**
**REUMATOID DISEASES**						
PsA, Ritchlin et al. (18)	168	3.7^*^	D>C	N.D.	N.D.	D>C
PsA, Colocci et al. (19)	49	0 [n.t.]^*^	D>C	55	50 [n.t.]	N.D.
PsA, Ikic et al. (20)	N.D.	N.D.	N.D.	120	40^*^	N.D.
RA, Miranda-Carus et al. (21)	100	5^*^	D>C	N.D.	N.D.	N.D.
RA and OA, Durand et al. (22)	N.D.	N.D.	N.D.	450^*^	350	N.S
RA, Ikic et al. (20)	N.D.	N.D.	N.D.	360^*^	40	N.D.
RA, Shang et al. (23)	N.D.	N.D.	N.D.	125^*^	75	N.S.
OA, Durand et al. (24)	N.D.	N.D.	N.D.	248^*^	210	D>C
AS, Caparbo et al. (25)	N.D.	N.D.	N.D.	700	750^*^	
Charcot's osteoarthropathy, Mabilleau et al. (26)	N.D.	N.D.	N.D.	96^*^	56 (diabetes) 21 (HC)	D>C
Acroosteolysis, Park et al. (27)	ND	ND	ND	142	18^*^	D>C
**OSTEOPOROSIS**						
Jevon et al. (28)	9	7	D>C	ND	ND	ND
D'Amelio et al. (17)	48	15^*^	D>C	50	40	N.D.
D'Amelio et al. (29)	145	5^*^	D>C	180	140	N.D.
Koek et al. (30)	N.D.	N.D.	N.D.	28	27	N.S.
**PERIODONTITIS**						
Brunetti et al. (12)	59	5^*^	D>C	70	62	N.D.
Tjoa et al. (31)	14	8	N.D.	14	15	
Herrera et al. (32)	17	8^*^	D>C	ns	ns	N.S.
**CANCER**						
Solid tumors, Roato et al. (33)	172	48^*^	D>C	161	132 [n.s.]	N.S.
Solid tumors, Roato et al. (34)	60	20^*^				
Prostate cancer, Roato et al. (35)	216	73^*^	N.D.	N.D.	N.D.	N.D.
Gastric, D'Amico et al. (36)	N.D.	N.D.	N.D.	90	40	N.S.
**CHRONIC LIVER DISEASE**						
Olivier et al. (37)	35	20^*^		45	37	D>C
**CROHN'S DISEASE**						
Oostlander et al. (38)	380	50^*^	0	ND	ND	
**CHRONIC KIDNEY DISEASE**						
Cafiero et al. (39)	20	1^*^	D>C	±40	40	N.D.
**TURNER'S SYNDROME**						
Faienza et al. (40)	49	5^*^	D>C	±42	±50	N.D.
**GAUCHER**						
Mucci et al. (41)	N.D.	N.D.	N.D.	±140	±75^*^	D>C
Reed et al. (42)	N.D.	N.D.	N.D.	±120	±40^*^	D>C
**PHENYLKETONURIA**						
Roato et al. (43)	149	91^*^	D>C	189	124^*^	ND

*N.D., not determined*;

* *, significantly different from disease; N.N., not significantly different from disease; D, Disease; C, control. Numbers of osteoclasts formed between studies are not comparable, since culture conditions differed between the studies*.

### Rheumatic Diseases

#### Rheumatoid Arthritis

Rheumatoid arthritis is a chronic inflammatory joint disease that is characterized by chronic synovitis and exaggerated local and systemic bone loss. It was established by Gravallese et al., that osteoclasts accumulate in the joints of RA patients (44) and that RANKL, the key osteoclastogenic mediator, is expressed locally in the joints of RA patients (45). Systemically, increased circulating osteoclast precursors have been reported in RA, which express key osteoclastogenic molecules on their surface (46, 47). Notably, when exposing similar numbers of peripheral CD14+ cells to osteoclastogenic conditions, more osteoclasts form in RA patients than in healthy controls (20), which is a strong argument that these peripheral osteoclast precursors are primed in the circulation. Moreover, elevated osteoclast formation in RA was shown to be correlated to local and systemic bone loss in RA (23). In addition, data showed that RA affects the longevity of osteoclasts, with a significantly lower number of osteoclasts undergoing apoptosis in RA compared to the healthy controls (22).

Enhanced osteoclastogenesis in RA essentially depends on T cells (48). Thus, in T-cell (CD3+ cells) depleted cultures, osteoclast precursors from RA patients show significantly lower spontaneous differentiation. Addition of exogenous RANKL to these cultures resulted in partial recovery of osteoclast formation. Together, these data indicate a crucial role for T-cells in osteoclast formation in RA, in line with earlier studies that demonstrated the activating role of T-cells in osteoclast formation (8, 49) which initiated osteoimmunology research. Miranda-Carus et al. (21) showed that T-cells present in peripheral blood play a major role in the formation of osteoclasts in RA patients. They interact with the osteoclast precursors of the monocyte/ macrophage lineage *in vitro*. An increased level of TNF-α, IL-1, IL-17, and RANKL was also found in the autologous T-cell/monocyte co-cultures derived from patients with early RA and established RA compared with controls (21) suggesting that these cytokines drive osteoclast differentiation in RA. Especially CD4+ T-cells play a role in osteoclastogenesis. OPG, anti-TNF-α and anti-IL-1 in this study, were shown to inhibit osteoclast formation (21).

#### Psoriatic Arthritis

Psoriatic arthritis (PsA) is a chronic inflammatory joint disease, which develops in about 30% of patients with psoriasis and is characterized by local bone erosions and systemic bone loss. TNFa and the interleukins 17 and −23 are key mediators of bone loss in PsA (50). In 2003, Ritchlin et al. (18) showed that the number of osteoclast precursors is increased in patients with PsA. In part, the development of osteoclasts in PsA could be independent from M-CSF or RANKL. This could be explained by a higher number of circulating OC precursors or by the ability of maturation of osteoclast precursors without supplementation with exogenous levels of M-CSF or RANKL. Ritchlin et al., also showed strong RANKL expression in synovial tissue of PsA patients as well as strong RANK expression in osteoclasts at areas with bone erosion. Fewer osteoclasts formed from blood of PsA patients after anti-tumor necrosis factor-α (TNF-α) treatment, indicating that TNF-α primes OC precursors in PsA (16). The original findings of Ritchlin et al., were also corroborated by Colucci et al., who showed that more TRAP+ multinucleated cells formed in cultures from PsA patients. Interestingly, supplementation of inflammatory cytokines abrogated these differences between PsA patients and controls (Table 1). This finding indicates an intrinsically higher osteoclastogenesis potential of blood from PsA patients. Increased osteoclast formation in PBMCs appears to be T-cell dependent, since the formation of osteoclasts was absent in T-cell-depleted PBMC cultures, which implies that T-cells are responsible for osteoclast formation. These results also suggest that blockade of RANKL and TNF-α might be used as an effective strategy for inhibiting enhanced osteoclastogenesis in PsA patients (19). Indeed such concept is supported by data on TNF inhibitors, which effectively inhibit osteoclast formation (51). Notably, aside from inflammation, PsA is strongly linked to obesity and the metabolic syndrome. In this context it is interesting that Xue et al., found an elevated value of certain adipokines, cytokines derived from adipose tissues, in the circulation of patients with PsA (52). Adipokines like leptin, adiponectin, chemerin, and omentin may not only play a role in inflammation but also in osteoclastogenesis in patients with PsA. Higher levels of leptin and omentin, for instance, positively correlated with the number of osteoclast precursors found in PsA patients, while adiponectin was negatively correlated with osteoclast precursors.

#### Osteoarthritis

In osteoarthritis (OA) (24), the key degenerative joint disease, it was found that the number of TRACP+ multinucleated osteoclasts is higher than in healthy controls providing an explanation for the sometimes bone erosive phenotype of OA. No significant difference was found between OA and healthy controls regarding the number of circulating CD14+ cells. However, osteoclasts from the OA patients were found capable of resorbing a significantly larger (4 times) area than the osteoclasts from the healthy controls. The higher number of osteoclast-like cells formed by the PBMCs from the osteoarthritis group compared to the PBMCs from the control group could be responsible for enhanced local bone resorption in OA.

Though strictly speaking not a rheumatoid disease, Charcot's arthropathy that is associated with diabetes co-incides with joint erosions, in particular of the foot (53). Charcot's arthropathy is associated with increased peripheral blood osteoclast formation, with more osteoclasts than in matched diabetes patients without Charcot's arthropathy is or healthy controls (26). A later study from the same group showed that higher numbers of CD14+ cells prevail in blood of patients with Charcot foot, concomitant with higher peripheral blood TNF-α levels (53).

#### Acroosteolysis

Also patients suffering from acroosteolysis, which is part of the systemic sclerosis disease spectrum, show increased osteoclast formation compared to healthy controls. In this context, the increased osteoclast formation is associated with higher VEGF levels in the peripheral blood (27). VEGF can substitute for M-CSF in driving osteoclast differentiation (54).

#### Ankylosing Spondylitis

Ankylosing spondylitis is an inflammatory rheumatic disease of the spine, which is characterized by loss of trabecular bone but periosteal apposition of cortical bone leading to bony spur formation sometimes leading to fusion of vertebra (55). Interestingly, ankylosing spondylitis is the only rheumatic disease where data showed that less osteoclasts form *in vitro* and where data on serum CTX levels show that lower overall bone resorption happens. Lower osteoclast numbers correlated with lower RANKL/OPG ratios. Furthermore, osteoclasts from ankylosing spondylitits patients were less prone to apoptosis (25) which has also been observed in other rheumatic diseases (24).

### Osteoporosis

Osteoporosis is a skeletal disease characterized by lower bone mass and micro-architectural deterioration of bone leading to an increased risk of fractures (56). Several indications show that this phenomenon is caused by a higher activity of osteoclasts due to an imbalance of the osteoclasts and osteoblasts, postmenopausal bone loss is triggered by estrogen deficiency that increased osteoclastogenesis through several pathways, and in particular by the activation of T cells that produce higher level of pro-inflammatory and pro-osteoclastogenic cytokines as TNFα and RANKL (57). Bone fractures are the severe consequence of osteoporosis and represent a major health problem in the increasingly older (58). Old patients experiencing a femoral fracture have a decreased life expectancy and may become care-dependent in half the survivors. The presence of a fragility fracture increases the risk of new fractures creating a “domino effect”: one vertebral fracture doubles the risk of subsequent femoral fracture within a year, the presence of vertebral fractures as well as of femoral fracture impair patients' quality of life and increase mortality.

In a first study comparing the peripheral blood osteoclast formation from patients and controls, similar numbers of osteoclasts formed, but the resorptive capacity was higher in osteoclasts from osteoporosis patients (28), suggesting that the peripheral OCp had the same osteoclastogenic potential, but were somehow primed to form more active osteoclasts (28). D'Amelio et al. (17, 57) investigated the osteoclast formation in osteoporosis. The first study compared the osteoclast formation in osteoporotic women compared to healthy controls, without adding M-CSF, TNF-α, or RANKL to the cultures (17). A higher number of osteoclasts was formed in the cultures from osteoporosis blood compared to healthy controls. After supplementation of M-CSF and RANKL the numbers of osteoclasts reached a same level in controls and patients. A significantly higher level of TNF-α and RANKL was found in the PBMC cultures of the osteoporotic group. Adding 1,25-OH vitamin D3 to the PBMCs cultures resulted in both groups in lower numbers of osteoclasts, but higher resorption. The lacunar resorption area was significantly higher in the osteoporotic patients group compared to the healthy subjects with and without the addition of 1,25-OH vitamin D3. Comparable results were reported in the second paper by the same group (29): higher levels of TNF-α and RANKL in the PBMC cultures and higher osteoclast formation in the osteoporotic group compared to the healthy subjects. Antibodies against TNF-α and RANKL decreased spontaneous osteoclast formation strongest in the osteoporotic group. Additionally this study demonstrated that the T-cells of osteoporosis patients play a key role in the osteoclastogenesis by increasing the TNF-α and RANKL production. It was shown that osteoclast formation was severely suppressed when depleting the T-cells from the PBMCs cultures. This indicates that T-cells play a crucial role in osteoclast formation and that they secrete cytokines necessary for osteoclast formation in osteoporosis. In a fourth study, no differences in osteoclast formation were found between controls and osteoporotic patients (30), but in this case only M-CSF and RANKL stimulated cultures were studied. This was in line with the previous studies (17, 28, 57) regarding the stimulated osteoclastogenesis, where addition of M-CSF and RANKL may shield possible differences.

### Periodontitis

Periodontitis is the inflammatory bone-destructive disease affecting the alveolar bone between teeth. It's individual susceptibility is driven by an oral bacterial dysbiosis, genetic factors (59) and life style (60). Currently, it is estimated that no <46% of Americans adults have moderate to severe periodontitis (61). When peripheral blood mononuclear cells (PBMC) from chronic periodontitis patients were cultured in the absence of M-CSF and RANKL, more osteoclast-like cells form from chronic periodontitis patients (12). Osteoclast-like cells in the control group were fewer and smaller. The addition of stimulating factors M-CSF and RANKL resulted in comparable numbers of osteoclast-like cells in control and periodontitis group. M-CSF and RANKL only triggered osteoclast formation in the control group, osteoclast numbers of the periodontitis group did not increase after cytokine treatment. The osteoclasts formed spontaneously from the PBMC cultures from the periodontitis patients and showed a significantly higher resorbtive activity compared to the controls. A T-cell dependent osteoclastogenesis was shown in this study, since the number of osteoclasts was low in the T-cell depleted unstimulated PBMCs cultures from the periodontitis patients. Addition of stimulating factors M-CSF and RANKL to these cultures led to a significantly higher formation of numerous large osteoclasts. An explanation for this finding is the overexpression of RANKL and TNF-α by T-cells, which was shown to be higher by the T-cells from the patient group. Addition of anti-RANKL and anti-TNF-α antibodies induced a dose dependent inhibition of the osteoclast formation in the periodontitis group (12). Also Tjoa et al., aimed to determine if there was a difference in osteoclast formation between the PBMC from patients suffering from chronic periodontitis and matched healthy controls (31). In this study, no differences were observed in unstimulated osteoclast formation between controls and periodontitis patients. A significant difference was shown however after the stimulation with M-CSF between control and diseased group. Larger and more multinucleated cells were found in the control group, whereas the patient group was insensitive to stimulation with M-CSF (31), suggesting that the OCp in this group were already primed in the circulation. In another study, it was shown that peripheral blood monocytes from periodontitis patients are more prone to differentiate into mature multinucleated osteoclasts (32). In other words, this study shows that these monocytes were primed in peripheral blood and prepared here for enhanced osteoclast formation. Only the stimulation with RANKL and not with M-CSF and RANKL gave significant differences between the periodontitis group and healthy controls regarding the osteoclast-like cells formed. A significantly higher level of M-CSF was found in the periodontitis group (32). This could be an explanation for the insensitive response of PBMC from periodontitis patients to stimulation with M-CSF in the study of Tjoa et al. (31), since the osteoclast precursors could be already pre-activated by higher M-CSF levels present in serum.

### Cancer That Metastasizes to Bone

#### Hematological Cancers

B cell multiple myeloma was studied by Colucci et al. who cultured 10 times more osteoclasts in the absence of added cytokines from myeloma blood than from control blood. As described repetitively above, this difference was no longer seen when the M-CSF and RANKL was added to these cultures. Colluci et al., found that peripheral blood contained upregulated OPG and RANKL levels. OPG co-precipitated with TRAIL, by which more RANKL became available. Autologous T-cells added to these osteoclasts, prolonged survival of osteoclasts (11).

#### Solid Tumor

Using blood from patients with bone metastasis from diverse primary tumors such as melanoma, lung, prostate, kidney, breast, and colon, it was found that these cultures gave rise to more osteoclast compared to controls when no cytokines were added (33). Addition of M-CSF and RANKL nullified this effect. OCp from tumor patients were further characterized and apart from CD14 and CD11, these precursors, and not in the control group, also expressed the osteoclast marker the vitronectin receptor αvβ3, which could be typical for precursors that are a bit further differentiated, indicating that the OCp in peripheral blood of cancer patients are already a step further in differentiation. Addition of OPG did not inhibit unstimulated osteoclast formation (33). It was shown that anti-TNF blocked osteoclast formation (33), which was recently confirmed in a separate study (51) Only T-cells from osteolysis patients expressed TNF-α, and also osteoclasts derived from cancer patients expressed TNF-α (33). The same group showed in subsequent years with a relatively large cohort of heterogeneous tumors (34) and in a group of prostate cancer (35) that most osteoclasts are formed without stimulation in patients with bone metastasis, compared to cancer patients without metastasis, and least osteoclasts formed from blood from controls. In both studies, a role of IL-7 was described. IL-7 serum levels were high in patients with metastasis, lower in sera from patients without and lower in control sera. T-lymphocytes could be identified as the source for IL-7, antibodies against IL-7 significantly inhibited osteoclast formation (34, 35). In a group of gastric cancer patients, no differences were observed in numbers of osteoclasts that differentiation from blood from patients with metastases, from patients without metastases and controls (36). An important difference with the other solid tumor studies was that only cytokine stimulated conditions were considered.

### Rarer Bone Diseases Associated With Increased Bone Loss

#### Diseases Associated With Osteopenia: Chronic Liver Disease, Crohn's Disease and Chronic Kidney Disease

Chronic liver disease can lead to osteoporosis, but not in all individuals with chronic liver disease. In attempt to explain this phenomenon, osteoclasts were cultured from blood of chronic liver disease patients with osteopenia, without osteopenia and matched healthy controls. More osteoclasts formed without adding osteoclastic cytokines M-CSF and RANKL from blood of osteopenic patients compared to non-osteopenic and healthy controls (37). Interestingly, and in line with what was found in the periodontitis study from the same group (31), only controls responded with higher osteoclast numbers when stimulated with M-CSF, whereas both osteopenic and non-osteopenic patients did not respond to addition of M-CSF. Serum levels M-CSF of both patient groups were significantly higher than controls, suggesting M-CSF priming of OCp in peripheral blood. Furthermore, number of osteoclasts cultured *in vitro*, correlated negatively with the lumbar spine density score (37).

Though Crohn's disease is an intestinal disease, it is associated with inflammatory flair-ups periods. Some of the patients develop osteoporosis, including those who are not on high levels of corticosteroids. In a study with patients in a quiescent disease stage, Oostlander et al. (38) were the first to describe the pre-stages of unstimulated osteoclast formation. It was shown that the formation of osteoclasts is preceded with a stage of cell clusters, the number of which correlate with the numbers of osteoclasts that form (38). In a similar approach as in a previous study (57), OCp were either cultured with purified autologous B-cells, T-cells or the combination or without. Osteoclasts only formed in combinations where T-cells were present, which also made part of the cell clusters that preceded osteoclast formation. More osteoclasts formed from OCp:T-cell cultures from Crohn's disease than from controls and correlated to IL-17 levels *in vitro*. TNF-α levels were highest in OCp+ T-cells, compared to T-cells alone (no secretion) or monocytes only (lower levels), indicating that the T-cell:OCp interaction induces TNF-expression (36), later confirmed by Moonen et al. (3). Interestingly, the diverse combinations of T-cells, B-cells, or T-Cells + B-cells did not affect control monocytes, indicating that the osteoclastogenesis driving T-cell activity is increased in Crohn's disease.

Bone disease in patients with chronic kidney disease (CKD) is a major clinical concern due to its prevalence and consequences that greatly impact patients quality of life (62). CKD patients may be affected by both higher and lower bone turnover disease, in patients with high bone turnover disease increased osteoclastogenesis is sustained by both increased in inflammatory and pro-osteoclastogenic cytokines and by increased PTH due to the decreased ability of kidney to hydroxylate vitamin D into its active form 1,25OHvitamin D. Patients with terminal kidney failure who are on dialysis, experience a chronically inflammatory state with often skeletal complications. Osteoclast formation was studied both without and cytokine stimulation (39). Osteoclast formation was lowest in controls, and higher in early chronic patients and higher in late chronic patients and highest in hemodialysis patients, similar correlations were seen with resorptive capacity. RANKL was expressed on T-lymphocytes in renal patients, not in controls. RANK-Fc can inactive RANKL, and when added it dose dependently decreased osteoclastogenesis, demonstrating that osteoclast formation was RANKL dependent (39).

#### Rare Diseases Associated With Bone Loss: Turner's Syndrome, Gaucher's Disease and Phenylketonuria

Patients with Turner's syndrome may present with decreased bone due to hypergonadism associated with the disease. Estrogen deficiency could be the course for such bone loss (40). Unstimulated osteoclast formation resulted in more osteoclasts that were more active, both in the group with high levels of follicle stimulating hormone (FSH) and with low levels. These osteoclasts resorbed more calcium phosphate. Osteoclast numbers from monocytes with addition of M-CSF and RANKL were similar between controls and patients. The increased unstimulated osteoclast formation in Turner's syndrome correlated with a lower percentage of osteoclastogenesis inhibitory CD4+CD25+ cells and a higher percentage of CD3+ NKT cells. The CD8+TNF-α+ cells were higher in Turner's syndrome, as well as the CD14+TNF-α+ monocytes. This skewness could contribute and be responsible for the increased osteoclast formation (40).

Gaucher's disease is a heritable disease with deficiency of the lysomal enzyme glucocerebrosidase, resulting in excess of glycosylceramide, which is then stored in high quantities in macrophages, concomitant with a disturbed immunological balance and cytokine secretion profiles (42). Approximately 3-fold more osteoclasts formed from Gaucher patient's PBMC cultured with M-CSF and RANKL. These osteoclasts were larger in size and number of nuclei and resorbed larger areas of bone. When distinguishing between active and non- active bone disease, the Gaucher patients with active bone disease formed more osteoclasts. Especially the Gaucher cultures were relatively independent of M-CSF (42), in line with data from periodontitis (31) and chronic liver disease patients (37). When control PBMC were cultured with inhibitors of glucocerebrosidase, increased numbers of osteoclast formed (42), indicating that this enzyme plays a key role in tempering osteoclast numbers. The increased osteoclast formation in Gaucher's disease was confirmed by Mucci et al. (41). Here, it was shown that Gaucher's disease blood contained a higher percentage of non-classical/inflammatory CD14+CD16+ cells compared to the classical CD14+CD16– monocytes. When culturing with an enzyme that replenishes the missing glucocerebrosidase, osteoclast numbers decreased only in Gaucher patients cultures, again indicating that glucocerebrosidase tempers osteoclast formation. T-cells from Gaucher's disease express more RANKL (41). Osteoclast cultures from controls were insensitive to OPG or anti-TNF-α treatment, whereas osteoclast numbers went significantly down in Gaucher's PBMC cultures that were treated with OPG or anti-TNF-α (41).

Phenylketonuria (PKU) is a rare, inherited disease with a defect in the synthesis of the amino acid phenylalanine. These patients have a hitherto not understood progressive bone impairment. Also with this disease, increased osteoclasts were cultured both without osteoclastogenesis stimulating cytokines and with these cytokines (43). As shown for Gaucher (41), the blood of PKU also contains a higher proportion of CD14+CD16+ monocytes, which co-express CD51/CD61, or αvβ3 integrin, that is typical for osteoclasts (63). The unstimulated osteoclastogenesis cultures contained increased levels of TNF-α and RANKL in PKU patients. Only RANK-Fc, that blocks RANKL activity, decreased osteoclast numbers in PKU cultures. PKU patients contained activated T- cells of the CD4+CD25+CD69+ signature, a cell type that was absent in controls.

## General Osteoclastogenesis Features of Inflammatory Bone Diseases

When summarizing the osteoclastogenesis capacity of the various inflammatory bone disease, several features become apparent (Table 1). Firstly, when taking together all 29 summarized studies, more osteoclasts formed, be it unstimulated or stimulated with M-CSF and RANKL. This was the case for 24 of the 29 studies. Secondly, in all cases where resorptive activity was determined, the osteoclasts from bone loss diseases were more active. Thirdly, osteoclasts can be cultured without exogenous addition of M-CSF and RANKL, only when cultured from PBMC or at least the addition of T-cells to OCp. These osteoclasts often displayed lytic activity of calcium phosphate coatings, but also bone resorption activity has been reported. Fourthly, those studies that have compared unstimulated and M-CSF and RANKL stimulated osteoclast formation, often found increased osteoclast formation in unstimulated cultures, whereas these differences were often not found any more when cultured with M-CSF and RANKL. This accounted for a variety of diseases, such as psoriatic arthritis (19), osteoporosis (17, 57), periodontitis (12, 31), multiple myeloma (11), solid tumors (33), chronic liver disease (37), kidney disease (39), and Turner's syndrome (40). This suggests that stimulation with unphysiological levels of M-CSF and RANKL may hide the intrinsically increased osteoclastogenic activities present in peripheral blood. Especially these studies with self-stimulatory osteoclastogenesis cocktails of T-cells and OCp can give us clues of common and disease specific determinants of increased osteoclast formation. Below, three common denominators which stood out when comparing the various inflammatory bone diseases, being (1) inflammatory mediators in serum, (2) the role of T-cells and (3) differential priming or skewness in monocyte distribution are worked out for the inflammatory bone diseases.

### Common Inflammatory Mediators in Serum Prepares OCp in the Circulation

A different priming whilst in the circulation by increased levels of pro-osteoclastogenic mediators could make monocytes more equipped to differentiate into osteoclasts. Most commonly described is the increased presence of TNF-α, see the above paragraph where it's presence is discussed in the context of T-cells, but also in serum from patients with Charcot's disease, where it co-incides with increased numbers of CD14+ cells (53). Another such pro-osteoclastogenic cytokine is M-CSF. Higher levels of M-CSF in the circulation have been described for periodontitis (32) and chronic liver disease (37). This could make PBMC from these patients less responsive to exogenous M-CSF (31, 37).

### A Common Role for T-Cells in Osteoclast Formation in the Various Inflammatory Bone Diseases

The general role of T- cells in osteoclast regulation (64), the role of osteoclast activating T-cells such as Th17 (65) and the regulatory role of Treggs (66) have been reviewed elsewhere. Here, we describe the T-cell findings in the context of inflammatory diseases with bone loss in the context of the core collection used for this review. When reviewing the above literature it is clear that T-cells are indispensable for spontaneous osteoclastogenesis: without T-cells, no osteoclasts form. This has been investigated for psoriatic arthritis (19, 48), osteoporosis (57), periodontitis (12) and Crohn's disease (38). The mechanism by which they adhere and stimulate OCp is likely by LFA-1: ICAM-1 interaction, since antibodies against LFA-1 interfere both with cell cluster formation and with osteoclast formation (38). TNF-α is most commonly reported as the cytokine secreted by T-cells in the various diseases. T-cells from patients with inflammatory bone loss diseases secrete more TNF-α, as shown for psoriatic arthritis (19), osteoporosis (57), periodontitis (12), and Turner's syndrome (40). Exclusively the T-cells from peripheral blood from patients with osteolytic solid tumors and not those without osteolytic tumors were reported to express detectable levels of TNF-α (34). Treating unstimulated osteoclastogenesis cultures with anti TNF-α agent infliximab reduces both cell cluster formation and osteoclast formation (51). Anti-TNF-α treatment also decreased osteoclast formation in patients with RA (18), periodontitis (12), and Gaucher's disease (41). T-cells from inflammatory bone diseases express more RANKL, as reported for RA (21), osteoporosis (57), and chronic kidney disease (39). Anti RANKL reduced osteoclast formation in Gaucher's disease more than in controls (41), and also in phenylketonuria patients (43), chronic kidney disease (39). Studies with RANKL-independent osteoclast formation were also reported (33, 37). In these studies, OPG or RANK-Fc did not affect osteoclast formation. Two osteoclastogenesis studies from solid tumors report spontaneous osteoclastogenesis stimulated by T-cell secreted IL-7 (34, 35). This has not been studied in the other inflammatory bone loss diseases. Finally, it has been reported for myeloma derived osteoclasts that autologous T-cells added to osteoclast cultures may prolong osteoclast survival (11).

### A Different Distribution of Monocytes Subtypes and Different Monocyte Priming in Inflammatory Diseases

A third commonality between the different diseases is that due to the inflammatory disease, a skewed distribution of monocytes that is better equipped to differentiate into osteoclasts populate the peripheral blood. The existence of different monocyte populations (2, 67) which distributions are then different between disease and controls, is indeed a very attractive explanation for the outcome of more osteoclasts in inflammatory disease. One way to achieve a relatively crude and likely heterogeneous populations of OCp is with CD14+ positive isolation. Studies that show more osteoclasts as outcome, where equal numbers of purified CD14+ monocytes were uses in disease vs. healthy controls, provide a first indication for a better equipment of these cells for osteoclast differentiation. This has been shown for RA and psoriatic arthritis (20) and for periodontitis (32). When comparing monocyte heterogeneity, two different criteria have been used in the articles assessed in this review: an approach based on CD14, CD11b, and the vitronectin receptor (VNR) expression and the more commonly used CD14/CD16 expression of monocytes. One study that has used CD14+ in conjunction with CD11b and the vitronectin receptor or αvβ3 showed that peripheral blood of osteolytic cancers contained more VNR+ monocytes, which correlated to higher osteoclast formation (33).

The much more commonly used classification of monocytes is based on their CD14 and CD16 expression. The CD14+CD16– are the classical monocytes that become the phagocytic cells in tissues and comprise the majority of blood monocytes, intermediate monocytes are CD14+CD16+ and are pro-inflammatory and play a role in wound healing, and the CD14+CD16++ cells are the non-classical monocytes that play a role in patrolling and fibrosis (2, 67). Initial description on either CD16– and CD16+, before the subsequent refinement of CD16+ monocytes into intermediate and non-classical monocytes (68) and also recent literature on the transcriptome of the classical and non-classical monocytes (69) suggest that the physiological osteoclasts derive from classical, CD14+CD16– monocytes. This was refined recently by Sprangers et al., who found that all three monocyte subtypes differentiate equally well on plastic, but that only the classical and intermediate ones form resorbing osteoclasts on bone (6). On bone slices, non-classical monocytes hardly differentiated and no resorption was observed. For some of the inflammatory bone loss diseases, however, several studies indicate that likely the CD16+ monocytes are important for osteoclast formation. A skew distribution compared to healthy controls was demonstrated for several inflammatory bone loss diseases. It was demonstrated for multiple myeloma, that CD14+CD16+ monocytes are predominant in active disease compared to smoldering disease. These cells gave rise to larger osteoclasts (70). Also in kidney disease, more CD14+CD16+ monocytes were found in peripheral blood, and exclusively more “inflammatory” monocytes expressed RANKL, providing the possibility for auto-stimulation (39). Patients with Gaucher's disease (41) and patients with phenylketonuria (43) have more CD14+CD16+ monocytes in peripheral blood than matched controls. All the above studies are association studies, where a higher percentage of CD14+CD16+ in blood of patients is associated with increased osteoclast formation. To prove that these cells indeed give rise to more osteoclasts, Chiu et al., have sorted the three subtypes of OCp from psoriatic arthritis patients and healthy controls (71). Interestingly, the CD16+ monocytes from controls gave rise to low levels of osteoclasts, whereas the CD16+ monocytes from patients gave rise to high numbers of osteoclasts. This suggests that apart from the common distinction of OCp with the CD14 and CD16 markers, other features have been acquired by psoriatic arthritis patients, making them more equipped to differentiate into osteoclasts. An experiment with healthy control OCps confirmed the relative inertness of CD16+ monocytes in healthy controls. When adding increasing numbers of CD16+ monocytes to a constant number of CD14+CD16– monocytes in an osteoclastogenesis experiment, there was no increase in osteoclast numbers, suggesting that under these conditions, only the CD14+CD16– monocytes contributed to the formation of a syncytium (72).

### Concluding Remarks on the Common Denominators for Increased Osteoclast Formation in Patients With Inflammation Related Bone Loss

**Figure 2:**
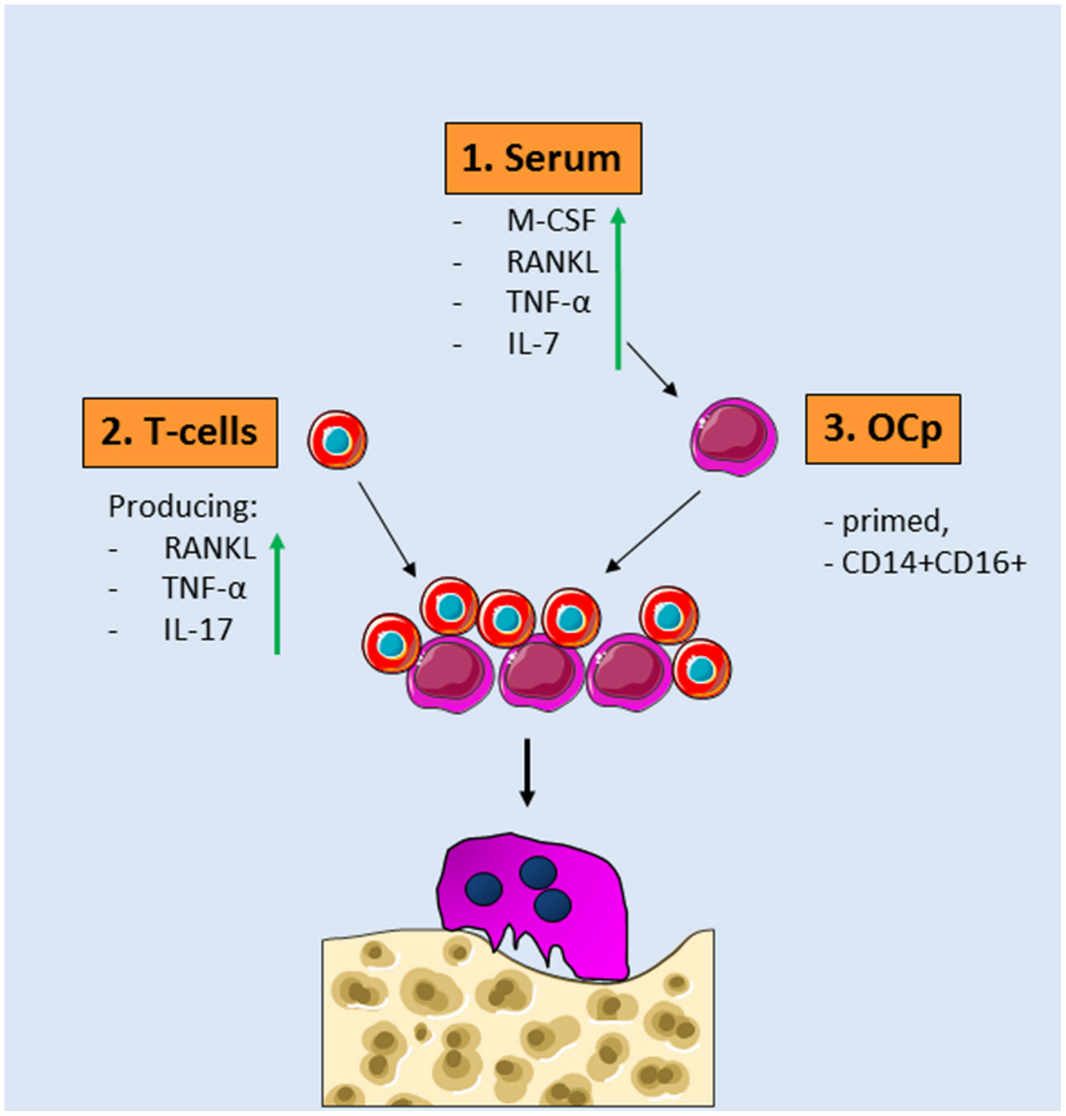
Common denominators for osteoclast formation in diseases with inflammatory bone loss. **1**. Serum of patients with inflammatory bone loss diseases contains more osteoclastogenesis stimulating factors such as RANKL, TNF-α, IL-7, and M-CSF. **2**. These serum factors prime OCp present in peripheral blood. These OCp are skewed toward more CD14+CD16+ cells. **3**. T-cells from patients with inflammatory bone diseases express more IL-17, RANKL, and TNF-α. When these different OCp and T-cells—both of them different from controls—are added together, more osteoclasts are formed that are more active in bone resorption.

In summary, this core collection of studies with well-matched bone-loss patient—healthy controls, unequivocally shows an increased osteoclast formation and activity in patients with inflammatory bone loss. In search for the conditions that give rise to this, several factors that are shared between diseases can be identified. First of all, the serum that surrounds OCp in the circulation is beneficiary for the differentiation of OCp. Second, T-cells that secrete TNF-α and RANKL are present in the circulation of patients. In light of the fact that inflammatory bone loss diseases may occur simultaneously, anti-TNF-α treatment could benefit more than one disease, as was shown for instance in rheumatoid arthritis patients receiving anti-TNF-α medication infliximab, who had lower periodontal indices. Finally, the patient blood may contain more OCp and differently primed OCp, probably containing the CD14+CD16+ phenotype rather than CD14+CD16–. On top of that, either disease specific or a general immunological stimulus has given the OCp from bone loss patients a profile to facilitate enhance osteoclast formation (Figure 2). Future research will likely uncover the barcodes of the OCps in the various inflammatory diseases associated with bone loss. This knowledge of the biological mechanisms underlying the alterations of monocytes and osteoclasts will likely reveal future therapeutic targets that will specifically target the immune system-steered osteoclast formation.

## Author Contributions

TV wrote most of the manuscript and coordinated feedback. IB performed literature search and drafted the initial parts of the manuscript. TK commented on earlier versions of the manuscript. GS critically evaluated the rheumatology part. IJ critically evaluated all stages of the manuscript. GS and PD put the manuscript in a clinical context. PD wrote the sections on osteoporosis.

### Conflict of Interest Statement

The authors declare that the research was conducted in the absence of any commercial or financial relationships that could be construed as a potential conflict of interest.
